# Awareness, knowledge, and attitudes related to HIV pre-exposure prophylaxis and other prevention strategies among physicians from Brazil and Mexico: cross-sectional web-based survey

**DOI:** 10.1186/s12913-022-07900-y

**Published:** 2022-04-22

**Authors:** Hamid Vega-Ramirez, Thiago S. Torres, Centli Guillen-Diaz, Cristina Pimenta, Dulce Diaz-Sosa, Kelika A. Konda, Alessandro Ricardo Caruso da Cunha, Rebeca Robles-Garcia, Marcos Benedetti, Brenda Hoagland, Daniel R. B. Bezerra, Carlos F. Caceres, Beatriz Grinsztejn, Valdilea G. Veloso, for the ImPrEP Study Group

**Affiliations:** 1 Center for Research in Global Mental Health, National Institute of Psychiatry Ramon de la Fuente Muñiz, Calzada México-Xochimilco 101, Col. San Lorenzo Huipulco, Alc. Tlalpan, 14370 Mexico City, Mexico; 2 Instituto Nacional de Infectologia Evandro Chagas, Fundação Oswaldo Cruz (INI-Fiocruz), Rio de Janeiro, RJ Brazil; 3 Ministry of Health, Brasilia, DF Brazil; 4 Center for Interdisciplinary Research in Sexuality, Health, and AIDS, Universidad Peruana Cayetano Heredia, Lima, Peru

**Keywords:** Pre-exposure prophylaxis, Post-exposure prophylaxis, HIV, Physicians, U=U slogan, Treatment as prevention, Brazil, Mexico, Latin America

## Abstract

**Background:**

In order to end the HIV epidemic by 2030, combination HIV prevention including pre-exposure prophylaxis (PrEP) should be widely available, especially for the most vulnerable populations. In Latin America and the Caribbean (LAC), only 14 out of 46 countries have access to PrEP. In Brazil and Mexico, PrEP has been provided at no cost through the Public Health System since 2017 and 2021, respectively. Thus, HIV physicians’ perspectives about PrEP and other prevention strategies may differ. This study aimed to compare awareness, knowledge, and attitudes related to PrEP and other prevention strategies among HIV physicians from Brazil and Mexico.

**Methods:**

Cross-sectional, web-based survey targeting physicians who prescribe antiretrovirals from both countries. Participants answered questions on socio-demographic, medical experience, awareness, knowledge, and attitudes towards PrEP and other HIV prevention strategies. We stratified all variables per country and compared frequencies using Chi-square, Fisher exact, and Wilcoxon-Mann-Whitney tests, as appropriate.

**Results:**

From January–October 2020, 481 HIV physicians were included: 339(70.5%) from Brazil, 276(57.4%) male, and median age was 43 years (IQR = 36–53). Awareness of PrEP did not differ between Brazil and Mexico (84.6%), while awareness of other prevention strategies, including post-exposure prophylaxis and new PrEP technologies, was higher in Brazil. More Brazilians perceived U=U as completely accurate compared to Mexicans (74.0% vs. 62.0%, *P* < .001). Willingness to prescribe PrEP was 74.2%, higher among Brazilians (78.2%, *P* = .01). Overall, participants had concerns about consistent access to PrEP medication and the risk of antiretroviral resistance in case of acute HIV infection or seroconversion. The main barriers reported were assumptions that users could have low PrEP knowledge (62.0%) or limited capacity for adherence (59.0%). Compared to Brazilians, Mexicans reported more concerns and barriers to PrEP prescription (all; *P* ≤ .05), except for consistent access to PrEP medication and the lack of professionals to prescribe PrEP (both; *P* ≤ .01).

**Conclusions:**

Although awareness of PrEP was similar in Brazil and Mexico, differences in knowledge and attitudes may reflect the availability and stage of PrEP implementation in these countries. Strengthening and increasing information on PrEP technologies and other HIV prevention strategies among HIV physicians could improve their comfort to prescribe these strategies and facilitate their scale-up in LAC.

**Supplementary Information:**

The online version contains supplementary material available at 10.1186/s12913-022-07900-y.

## Background

The annual number of new HIV diagnoses has not changed since 2010 (100,000 new infections), with 2.1 million people living with HIV in Latin America and the Caribbean (LAC) by the end of 2020 [1]. Gay, bisexual, men who have sex with men (MSM) and transgender women (TW) remain the most affected populations in the region [1]. In order to end the HIV epidemic by 2030, combination HIV prevention should be widely available especially for the most vulnerable populations, with strategies including behavioral, biomedical, and structural approaches based on human rights, and community-based interventions, such as gender-affirming approaches [2, 3]. Pre-exposure prophylaxis (PrEP) is an effective biomedical prevention strategy to prevent new HIV infections [4, 5]. The World Health Organization strongly recommends the incorporation of daily oral PrEP with tenofovir disoproxil fumarate 300 mg and emtricitabine 200 mg (TDF/FTC) into combination HIV prevention package since 2016 [6]. Nevertheless, by the end of June 2021 only 14 out of 46 LAC countries had access to PrEP, mostly through private clinics, nongovernmental organizations, pilot studies, or implementation/demonstration projects [7].

Brazil and Mexico host half of LAC population and have the largest gross domestic product in the region [8]. Both countries provide combination HIV prevention at no cost through the public health system, including condoms, post-exposure prophylaxis (PEP), test and treat, and antiretroviral (ARV) treatment for all people newly diagnosed with HIV [9, 10]. However, there is different PrEP availability in the two countries [7, 9, 10]. Brazil participated in the iPrEX clinical trial (2007–2010) [11] and conducted the PrEP Brasil Demonstration Study to evaluate acceptability, retention, and adherence of PrEP among MSM and TW (2014–2016) [12]. These two experiences paved the way for Brazil to start providing PrEP at no cost through its national public health service (Brazilian Unified Health System *– SUS*, in Portuguese) since 2017 [13]. By October 2021, 47,821 Brazilians had initiated PrEP and 27,236 were using PrEP [14]. The Implementation PrEP project (ImPrEP) aims to generate evidence on the acceptability, feasibility, and cost-effectiveness of PrEP among MSM and TW in Brazil, Mexico and Peru, including a large PrEP demonstration study conducted from 2018 to 2021 [15]. ImPrEP was the first opportunity for Mexico to provide PrEP in three cities (Mexico City, Guadalajara and Puerto Vallarta), with 2445 participants under follow-up by the end of June 2021 [16]. In 2021, the Mexican National HIV Program (CENSIDA, in Spanish) and the social security health system launched national pilot PrEP programs [17, 18]. PrEP availability and demand creation including awareness and knowledge of users and health care professional are fundamental for PrEP scale-up among populations vulnerable to HIV. Until 2020, only HIV physicians in the public sector could prescribe PrEP in these two countries. The different stages of PrEP availability and implementation in Brazil and Mexico could lead to differences in HIV physicians’ perspectives about PrEP and other prevention strategies.

Awareness and willingness to prescribe PrEP may increase depending on PrEP availability in the health systems [19]. Nevertheless, physicians may have concerns or perceived barriers about prescribing PrEP [20]. Low knowledge about PrEP, lack of time, cost, antiretroviral resistance, the *purview paradox* (the belief that PrEP prescription is beyond one’s clinical domain), interpersonal stigma, anticipated risk compensation, and PrEP adherence were the main reported concerns of health care physicians in the USA [20–23]. Low willingness to prescribe PrEP has been associated with providers’ racial bias or prejudice about key populations behavior, especially in their PrEP adherence capacity [24, 25]. Studies in LAC reveal variations in awareness and willingness to prescribe PrEP among general practitioners or physicians with a medical specialty. In 2015, in Guatemala, 69% of internal medicine and infectious disease trainees reported awareness of PrEP, and 87% had willingness to prescribe it [26]. In Brazil, a study conducted between 2016 and 2017 showed that 75% of infectious disease physicians were aware of PrEP, and between 63 and 69% reported willingness to prescribe PrEP for MSM or sex workers with inconsistent condom use [27]. However, such information is not available for Mexico and for Brazil after PrEP implementation in December 2017.

As part of the ImPrEP project, we conducted a web-based survey among HIV physicians from Brazil and Mexico to understand awareness, knowledge, experience, and attitudes related to PrEP and other HIV prevention strategies, as well as to compare both countries considering the differences in PrEP implementation stage.

## Methods

### Study design

This was a cross-sectional web-based survey targeting HIV physicians who prescribe ARV from Brazil and Mexico. In both countries, most ARV prescriptions, including PrEP, are performed by physicians working in the public health system (either general practitioners or infectious diseases specialists). HIV physicians who signed electronic informed consent were included. We excluded participants who previously participated in the survey. We used Alchemer^®^ (Brazil) and SurveyMonkey^®^ (Mexico) for programming the questionnaire. The survey was designed based on previous studies [21, 23, 26, 27], and consisted of 37 questions in 22 pages. Participants could only answer questions on one page after completing all items on the previous page. The items related to perceived barriers, concerns or attitudes had a 4-point Likert scale to avoid intermediate options and reduce social desirability bias [28]. We piloted the questionnaire to a small sample of physicians with experience in ARV prescription in both countries. The research team discussed the post-pilot suggestions and adjusted items as needed.

In Brazil, the survey was conducted between January 28 and October 20, 2020. The questionnaire link was sent by e-mail using Mailchimp^®^ to all HIV physicians registered at *Siclom* (Brazilian National System for antiretroviral prescription and dispensation) and at the Federal Medicine Council (CFM). Those who did not initially respond were sent up to two additional emails. In Mexico, we sent weekly e-mails to HIV physicians between March 15 and September 4, 2020. Twenty-seven out of 32 State HIV Representatives from Mexico provided a list containing 267 HIV physician e-mail contacts.

### Variables

#### Socio-demographic and medical experience

We collected the following demographic characteristics: age (stratified in 26 to 34, 35 to 49, ≥50 years; and provided as median and interquartile range [IQR]), gender (male/female), race/skin color (White, Mixed [*Mestizo* in Mexico and Pardo in Brazil], Asian, Black and Indigenous), region of residence (Brazil: North, Northeast, Central-west, Southeast, and South; Mexico: Northeast, North Centre, South Centre, South, West, and East) and living in metropolitan area of State capitals (yes/no). Participants were asked about their medical experience: infectious diseases specialist (yes/no), number of years as medical doctor (MD; ≤5, 6–10, 11–15, 16–20, and > 20 years), and number of patients living with HIV under follow-up (None, 1–19, 20–49, ≥50).

#### Awareness of combination HIV prevention strategies including new PrEP technologies**,** and comfortableness on prevention counseling

We assessed awareness of combination HIV prevention and PrEP with two separate questions: “Have you ever heard of the combination HIV prevention concept (or PrEP)?”, using a 4-Likert scale for response options (*Not at all* to *Very much*). For the analysis, we considered participants who responded *Much* or *Very much* as aware. We provided a pre-existing list of other prevention strategies (ie, condoms, treatment as prevention, serosorting, etc) including new PrEP technologies, such as event-driven PrEP and cabotegravir injection [29, 30], and asked HIV physicians to choose all strategies they had previously heard about.

Participants were asked if they would feel comfortable performing activities related to HIV and sexual transmitted infections (STI) prevention (ie, discussing sexual behavior, requesting STI exams, etc). We presented possible answers in a 4-Likert scale (*Completely uncomfortable* to *Completely comfortable*) and participants were considered comfortable to perform such activities if they answered *Completely comfortable* or *Comfortable*. Participants were also asked about their awareness of the different PrEP technologies, such as TDF/FTC daily dose and cabotegravir injection (yes/no) [29, 30].

#### Knowledge and attitudes regarding PrEP, PEP and U=U slogan

HIV physicians answered whether they had knowledge of PrEP and PEP national guidelines or had ever received any training, prescribed, or referred a client to receive PrEP or PEP (yes/no). We also asked participants their willingness to prescribe PrEP and in which context or setting they had previously prescribed it: private office, demonstration studies, clinical trials and/or SUS (only for Brazilian participants) (all yes/no). For PEP, we asked about reasons for PEP prescription (occupational, sexual violence, and consensual sex; multiple options were available). Perceived accuracy of the undetectable equals untransmittable (U=U) slogan was assessed following previous studies (completely accurate vs. not) [31, 32]. Participants were also asked if they had ever been trained on the U=U (yes/no).

#### Populations who would benefit from PrEP, healthcare services that should offer PrEP, and reasons for not offering PrEP

We asked HIV physicians which populations would benefit from PrEP from a pre-existing list and which healthcare services should offer PrEP (HIV/STI clinics, specialized clinics, family clinics, private clinics/hospitals and primary care); possible answers were *yes*/*no* and multiple answers were permitted. We also asked about reasons PrEP should not be offered: “Public PrEP will reduce the budget for antiretroviral treatment”; “Behavioral interventions should be prioritized instead of PrEP”; “Low demand of PrEP users to maintain PrEP as public policy”; “I think PrEP should not be provided by public services”. For each of these items, responses were gathered using a 4-Likert scale (*Strongly disagree* to *Strongly agree*), *Strongly agree* and *Agree* responses were considered as *yes*.

#### Perceived concerns and barriers to prescribing PrEP

We provided a pre-existing list of concerns about prescribing PrEP (for instance, consistent access to PrEP medication and risky behavior increase) with possible answers in a 4-Likert scale (*Not concerned to Extremely concerned*); responses *Somewhat*/*Extremely concerned* were considered as *yes* for analysis. We also provided a pre-existing list of barriers to prescribe PrEP, with possible answers in a 4-Likert scale (*Not a barrier* to *Strong barrier*); responses *Moderate*/*Strong barrier* were considered as *yes*.

### Ethics and consent to participate

This study was approved by the Instituto Nacional de Infectologia Evandro Chagas, Fundação Oswaldo Cruz (INI-Fiocruz) Institutional Review Board (CAAE: 94050418.4.0000.5262) in Brazil and the Research Ethics Committee of the National Institute of Psychiatry Ramón de la Fuente Muñiz (CEI/C/038/2018) in Mexico. We did not collect participants’ identification or provide any incentives.

### Statistical analysis

We used only completed surveys for analysis and we described all study variables frequencies in total and for each country (total number of responses and percentages considering sample size). We compared responses between HIV physicians from Brazil and Mexico using Chi-square and Fisher exact test for categorical variables and Wilcoxon-Mann-Whitney for the continuous variable (age), as appropriate. Questions contained response options *I do not want to answer,* and *I do not know*, which were considered as missing data for analysis and not included in the frequency calculation. The items related to perceived barriers, concerns or attitudes scales were presented in absolute numbers and frequencies; scales were not developed to provide scores. Differences between countries were considered using a threshold *P* ≤ .05 for statistical significance. All analyses were performed using Stata/IC 15. Datasets generated and analyzed in this study are available (Additional file 1).

## Results

Of 704 participants who accessed the survey, 674 (95.7%) acknowledged informed consent and 541 (76.8%) completed the survey. Of these, 11.1% (60/541) reported previous participation in the study and were excluded. Our final sample was composed of 481 HIV physicians, 339 (70.5%) from Brazil and 142 (29.5%) from Mexico. Median age was 43 (IQR 36–53) years; most were male (279/481, 57.4%), self-identified as White (303/481, 63.9%), lived in metropolitan areas of state capitals (327/481, 68%), and were infectious disease specialists (333/481, 69.4%). Compared to Mexico, more HIV physicians from Brazil were younger (26–34 years; 22.3% vs. 12%; *P* = .01), White race (81.2% vs 22.3%; *P* < .001), infectious disease specialists (79.1% vs 46.1%; *P* < .001) and more experienced as MD (> 20 years; 44.2% vs 36.6%; *P* = .05). Conversely, Mexican physicians had more patients living with HIV under follow-up (≥50 patients; 91.3% vs 50.9%; *P* < .001) (Table 1). Most of the Brazilian HIV physicians reported living in the Southeast (55.4%), followed by South (19.2%), Northeast (13.9%), Central West (7.4%), and North (4.1%). For Mexico, most were from the South region (43.7%) followed by Northwest (23.2%), East (11.3%), West (9.2%), Northeast (6.3%), North Centre (5%), and South Centre (1.4%).

**Table 1:** Socio-demographics and medical experience of HIV physicians from Brazil and Mexico, 2020

	Total	Brazil	Mexico	*P* value^a^
	(*N* = 481) n (%)	(*N* = 339; 70.5%) n (%)	(*N* = 142; 29.5%) n (%)	
**Age (years)**				
26–34	90 (18.7)	73 (22.3)	17 (12)	.01
35–49	229 (47.6)	148 (45.3)	81 (57)	
≥ 50	162 (33.7)	106 (32.4)	44 (31)	
Median (IQR)	43 (36–53)	43 (35–54)	44 (38–51)	.75^b^
**Gender**				.48
Male	276 (57.4)	198 (58.4)	78 (54.9)	
Female	205 (42.6)	141 (41.6)	64 (45.1)	
**Race/skin color**				<.001^c^
White	303 (63.9)	272 (81.2)	31 (22.3)	
Mixed	152 (32.1)	51 (15.2)	101 (72.7)	
Asian	9 (1.9)	9 (2.7)	0 (0)	
Black	4 (0.8)	3 (0.9)	1 (0.7)	
Indigenous	6 (1.3)	0 (0)	6 (4.3)	
**Live in metropolitan area of state capitals**				.23
Yes	327 (68.0)	236 (69.6)	91 (64.1)	
No	154 (32.0)	103 (30.4)	51 (35.9)	
**Infectious disease specialist**				<.001
Yes	333 (69.4)	268 (79.1)	65 (46.1)	
No	147 (30.6)	71 (20.9)	76 (53.9)	
**Number of years as MD**^**d**^				.05
≤ 5	38 (7.9)	29 (8.6)	9 (6.4)	
6–10	81 (16.8)	59 (17.4)	22 (15.5)	
11–15	82 (17.1)	57 (16.8)	25 (17.6)	
16–20	78 (16.2)	44 (13)	34 (23.9)	
> 20	202 (42.0)	150 (44.2)	52 (36.6)	
**Number of patients living with HIV under follow-up**				<.001^c^
None	26 (5.5)	26 (7.8)	0 (0)	
1–19	65 (13.8)	56 (16.8)	9 (6.5)	
20–49	85 (18.0)	82 (24.6)	3 (2.2)	
≥ 50	296 (62.7)	170 (50.9)	126 (91.3)	

^a^Chi-square test

^b^Wilcoxon-Mann-Whitney test

^c^Fisher’s exact test

^d^MD: Doctor of Medicine

Overall, awareness of PrEP and other prevention strategies varied from 48.4% to 87.1% for cervical exams and PEP, respectively (Table 2). Awareness of PrEP was 84.6%, with no difference between countries (*P* = .25). Nevertheless, Brazilian HIV physicians were more aware of most prevention strategies including new PrEP technologies than their Mexican counterparts (*P* ≤ .05), except for daily oral TDF/FTC or tenofovir alafenamide/emtricitabine (TAF/FTC) (*P* ≥ .63). Most HIV physicians reported being comfortable performing all HIV/STI prevention activities evaluated with no difference between countries (*P* ≥ .20), except risk-reduction counseling, which was higher among Mexican compared to Brazilian physicians (99.3% vs 93.5%; *P* < .01).

**Table 2:** Awareness of PrEP and other prevention strategies, and comfort with HIV/STI procedures among HIV physicians from Brazil and Mexico, 2020

	Total	Brazil	Mexico	*P* value^a^
	(*N* = 481) n (%)	(*N* = 339; 70.5%) n (%)	(*N* = 142; 29.5%) n (%)	
**Awareness of PrEP and other prevention strategies (yes)**				
PEP	419 (87.1)	302 (89.1)	117 (82.4)	.05
Combination HIV prevention concept	407 (84.6)	294 (86.7)	113 (79.6)	.05
Condoms and lubricants	407 (84.6)	303 (89.4)	104 (73.2)	<.001
PrEP	407 (84.6)	291 (85.8)	116 (81.7)	.25
Regular HIV/STI testing	393 (81.7)	299 (88.2)	94 (66.2)	<.001
Mother to child transmission	371 (77.1)	286 (84.4)	85 (59.9)	<.001
Knowledge of partner serology	356 (74)	275 (81.1)	81 (57)	<.001
Treatment as prevention	347 (72.1)	280 (82.6)	67 (47.2)	<.001
Vaccination for HAV^b^, HBV^c^ and HPV^d^	335 (69.7)	270 (79.6)	65 (45.8)	<.001
Cervical exams	233 (48.4)	185 (54.6)	48 (33.8)	<.001
**Awareness of PrEP Technologies (yes)**				
Daily oral with TDF/FTC^e^	444 (92.3)	313 (92.3)	131 (92.3)	.98
Event-Driven PrEP with TDF/FTC^e^	262 (54.5)	212 (62.5)	50 (35.2)	<.001
Daily oral with TAF/FTC^f^	246 (51.1)	171 (50.4)	75 (52.8)	.63
Cabotegravir injection	202 (42)	180 (53.1)	22 (15.5)	<.001
Vaginal ring with antiretroviral	156 (32.4)	120 (35.4)	36 (25.4)	.03
Microbicides	104 (21.6)	82 (24.2)	22 (15.5)	.03
Implants with antiretroviral	97 (20.2)	81 (23.9)	16 (11.3)	<.01
Monoclonal antibodies	61 (12.7)	57 (16.8)	4 (2.8)	<.001
**Comfort with HIV/STI prevention procedures (yes)**				
Request HIV exam	477 (99.2)	337 (99.4)	140 (98.6)	.36
Request STI exams	473 (98.3)	335 (98.8)	138 (97.2)	.20
Evaluation of sexual risk behavior	458 (95.2)	324 (95.6)	134 (94.4)	.57
Risk-reduction counseling	458 (95.2)	317 (93.5)	141 (99.3)	<.01
Discuss sexual behavior	454 (94.4)	320 (94.4)	134 (94.4)	.99
Discuss sexual orientation	453 (94.2)	317 (93.5)	136 (95.8)	.33
Provide HIV+ result	441 (91.7)	310 (91.5)	131 (92.3)	.77
Evaluation of PrEP eligibility	432 (89.8)	301 (88.8)	131 (92.3)	.25
U=U^g^ counseling	387 (80.5)	274 (80.8)	113 (79.6)	.75
Request HIV acute infection test^h^	333 (69.2)	333 (98.2)	–	N/A^i^
Evaluation of PEP eligibility^h^	320 (66.5)	320 (94.4)	–	N/A

^a^Chi-square tests for all comparisons, except for *Monoclonal antibodies* (Fisher’s exact test)

^b^*HAV* Hepatitis A virus

^c^*HBV* Hepatitis B virus

^d^*HPV* Human papilloma virus

^e^*TDF/FTC* Tenofovir disoproxil fumarate / emtricitabine

^f^*TAF/FTC* Tenofovir alafenamide / emtricitabine

^g^*U=U* Undetectable equals untransmittable

^h^This question was not available in Mexico

^i^*N/A* Not applicable

**Fig. 1:**
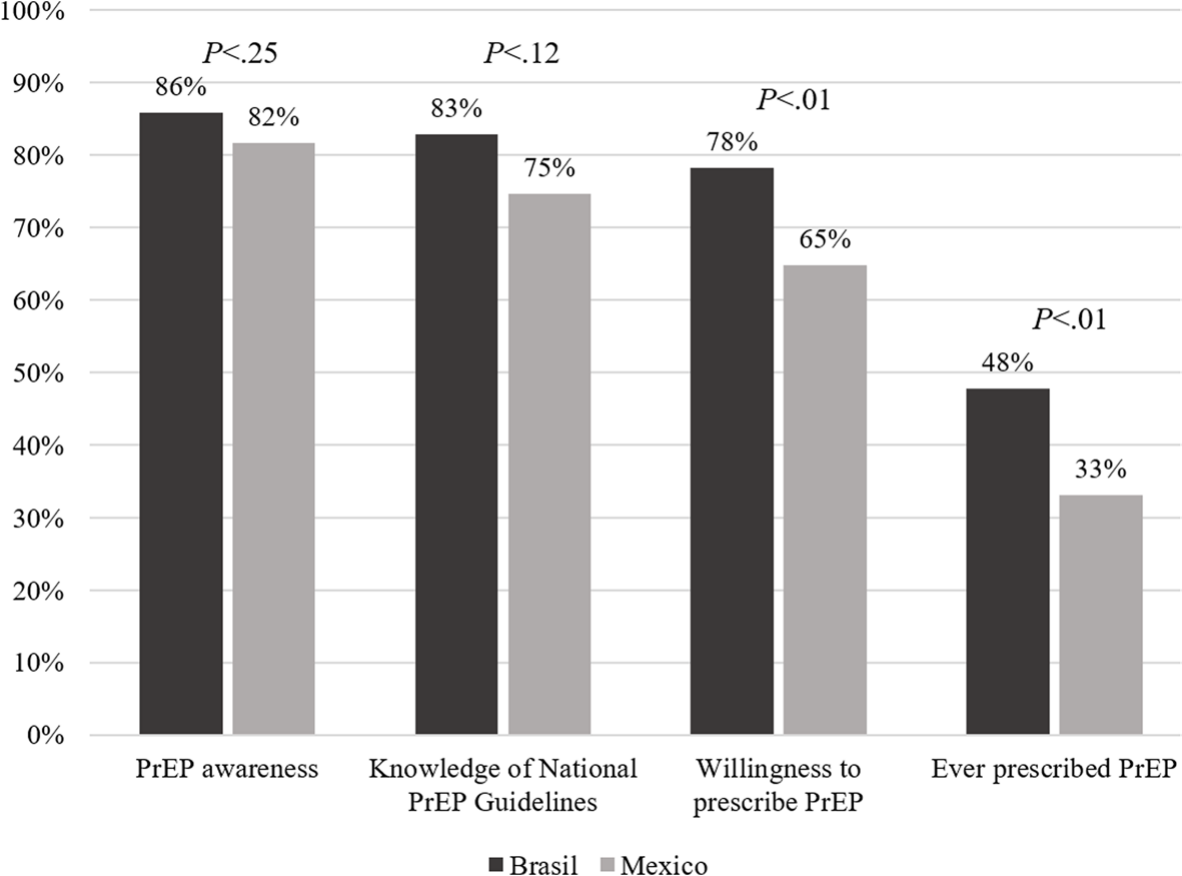
PrEP cascade experience (from awareness to prescription) differences between HIV physicians from Brazil and Mexico, 2020.^a^Adapted from Petroll AE, Walsh JL, Owczarzak JL, McAuliffe TL, et al. PrEP Awareness, Familiarity, Comfort, and Prescribing Experience among US Primary Care Providers and HIV Specialists. AIDS Behav 2017;21(5):1256–1267

Most HIV physicians from both countries knew about the national PrEP guidelines, with no differences between countries (*P* = .12) (Table 3). A higher proportion of Brazilian compared to Mexican physicians were willing to prescribe PrEP (78.2% vs 64.8%; *P* < .01), previously referred a patient to receive PrEP (72% vs 20.6%; *P* < .001) and had previous experience on prescribing PrEP (49.1% vs 33.3%; *P* < .01), while more Mexicans reported prescriptions at private offices (66% vs 45.1%; *P* = .01). Figure 1 provides the PrEP cascade experience (from awareness to prescription) differences between HIV physicians from Brazil and Mexico. More Brazilians than Mexicans knew about the national PEP guidelines (95.9% vs 83.1%; *P* < .001), previously referred a client to receive PEP (87.3% vs 44.4%; *P* < .001) and reported previous experience on prescribing PEP (92.6% vs 69.7%; *P* < .001), including all reasons for prescribing PEP. In Brazil, more HIV physicians perceived the U=U slogan completely accurate than in Mexico (74.0% vs 62%; *P* < .001), while more Mexicans had been previously trained in U=U (62% vs 38.6%; *P* < .001).

**Table 3:** Knowledge and attitudes regarding PrEP, PEP and U=U among HIV physicians from Brazil and Mexico, 2020

	Total	Brazil	Mexico	*P* value^a^
	(*N* = 481) n (%)	(*N* = 339; 70.5%) n (%)	(*N* = 142; 29.5%) n (%)	
**PrEP (yes)**				
Knowledge of national PrEP guidelines	387 (81.1)	281 (82.9)	106 (76.8)	.12
Willingness to prescribe PrEP	357 (74.2)	265 (78.2)	92 (64.8)	<.01
Ever referred a patient to receive PrEP	271 (57.7)	244 (72)	27 (20.6)	<.001
Ever trained in PrEP	139 (29)	93 (27.4)	46 (32.6)	.25
Ever prescribed PrEP	209 (44.4)	162 (49.1)	47 (33.3)	<.01
Site of PrEP prescribing				
Private office	104 (49.8)	73 (45.1)	31 (66.0)	.01
Demonstration studies	33 (15.8)	30 (18.5)	3 (6.4)	.06
Clinical trials	18 (8.6)	16 (9.9)	2 (4.3)	.38
SUS^**b**^	116 (24.1)	116 (71.6)	–	N/A^d^
**PEP (yes)**				
Knowledge of national PEP guidelines	418 (91.9)	300 (95.9)	118 (83.1)	<.001
Ever prescribed PEP	413 (85.9)	314 (92.6)	99 (69.7)	<.001
Ever referred a patient to receive PEP	359 (76.6)	296 (87.3)	63 (44.4)	<.001
Ever trained in PEP	209 (43.5)	148 (43.7)	61 (43)	.89
Reasons for PEP prescription				
Occupational	331 (80.1)	288 (91.7)	43 (43.4)	<.001
Consensual sex	278 (67.3)	252 (80.3)	26 (26.3)	<.001
Sexual violence	275 (66.6)	231 (73.6)	44 (44.4)	<.001
**U=U**^**c**^ **(yes)**				
Perceived U=U slogan as completely accurate	339 (70.5)	251 (74.0)	88 (62.0)	<.001
Ever trained in U=U	219 (45.5)	131 (38.6)	88 (62.0)	<.001

^a^Chi-square tests for all comparisons, except for *Site of PrEP prescribing* (Fisher’s exact test)

^b^SUS: Brazilian Unified Health System (in Portuguese)

^c^*U=U* Undetectable equals untransmittable

^d^*N/A* Not applicable

HIV physicians from both countries reported that MSM, sex workers, transgender people, and partners in sero-discordant relationships would be the populations benefiting the most by PrEP (Table 4). Conversely, more Brazilians than Mexicans agreed that PrEP would benefit almost all populations evaluated (for all, *P* ≤ .001) except for young adults or adolescents and MSM (*P* = .46). More Brazilians than Mexicans reported that PrEP should be offered in specialized clinics (79.9% vs 49.3%; *P* < .001) and private clinics/hospitals (40.4% vs 28.9%; *P* = .02), while more Mexicans reported it should be offered on HIV/STI clinics (88% vs 79.7%; *P* = .03). More Mexicans than Brazilians agreed with all reasons evaluated for not offering PrEP (for all, *P* ≤ .01); the reason most frequently reported for not offering PrEP in both countries was the prioritization of behavioral interventions instead of PrEP (54.3%, overall).

**Table 4:** Populations who would benefit from PrEP, Healthcare Services that should offer PrEP, and Reasons PrEP should not be offered according to HIV physicians from Brazil and Mexico, 2020

	Total	Brazil	Mexico	*P* value^a^
	(*N* = 481) n (%)	(*N* = 339; 70.5%) n (%)	(*N* = 142; 29.5%) n (%)	
**Populations who would benefit from PrEP (yes)**				
Gay, bisexual, and other men who have sex with men (MSM)	426 (88.6)	305 (90)	121 (85.2)	.13
Sex workers	422 (87.7)	327 (96.5)	95 (66.9)	<.001
Transgender people	388 (80.7)	286 (84.4)	102 (71.8)	.001
Partners in a sero-discordant relationship	363 (75.5)	271 (79.9)	92 (64.8)	<.001
Injectable drug users	291 (60.5)	227 (67.0)	64 (45.1)	<.001
Young adults or adolescents	228 (47.4)	157 (46.3)	71 (50)	.46
Non-injectable drug users	188 (36.1)	150 (44.2)	38 (26.8)	<.001
**Healthcare services that should offer PrEP (yes)**				
HIV/STI clinics	395 (82.1)	270 (79.7)	125 (88)	.03
Specialized clinics	341 (70.9)	271 (79.9)	70 (49.3)	<.001
Family clinics	191 (39.7)	143 (42.2)	48 (33.8)	.09
Private clinics/hospitals	178 (37)	137 (40.4)	41 (28.9)	.02
Primary care	172 (35.8)	124 (36.6)	48 (33.8)	.56
**Reasons PrEP should not be offered (yes)**				
“Behavioral interventions should be prioritized instead of PrEP”	261 (54.3)	148 (43.7)	113 (79.6)	<.001
“Public PrEP will reduce the budget for antiretroviral treatment”	202 (42)	120 (35.4)	82 (57.8)	<.001
“I think PrEP should not be provided by public services”	49 (10.2)	26 (7.7)	23 (16.2)	<.01
“Low number of PrEP users to maintain PrEP as public policy”	43 (8.9)	15 (4.4)	28 (19.7)	<.001

^a^Chi-square test

Perceived barriers and concerns to prescribe PrEP varied across countries (Table 5). Overall, more Mexican HIV physicians reported concerns and barriers to prescribe PrEP than Brazilians (for all, *P* ≤ .05), but more Brazilians reported concerns related to consistent access to PrEP medication (82.9% vs 68.3%; *P* < .001) and barriers related to lack of professionals to prescribe PrEP (62% vs 46.5%; *P* < .01).

**Table 5:** Perceived concerns and barriers to prescribe PrEP among HIV physicians from Brazil and Mexico, 2020

	Total	Brazil	Mexico	*P* value^a^
	(*N* = 481) n (%)	(*N* = 339; 70.5%) n (%)	(*N* = 142; 29.5%) n (%)	
**Concerns (yes)**				
Consistent access to PrEP medication	379 (78.6)	281 (82.9)	98 (68.3)	<.001
ARV^b^ resistance in case of acute HIV infection or seroconversion	362 (75.1)	243 (71.7)	119 (83.2)	<.01
Risky behavior increasal	361 (74.9)	238 (70.2)	123 (86)	<.001
Users need to take a drug everyday	361 (74.9)	255 (75.2)	106 (74.1)	.80
Risk of ARV drug resistance	326 (67.6)	215 (63.4)	111 (77.6)	<.01
Severe adverse effects	270 (56)	179 (52.8)	91 (63.6)	.03
Mild adverse effects	204 (42.3)	131 (38.6)	73 (51.1)	.01
Limited availability of ARV for people living with HIV	266 (55.2)	177 (52.2)	89 (62.2)	.04
PrEP efficacy	177 (36.7)	112 (33)	65 (45.5)	.01
**Barriers (yes)**				
Users have low PrEP knowledge	298 (62.0)	198 (58.4)	100 (70.4)	.01
Users have limited capacity for PrEP adherence	284 (59.0)	188 (55.5)	96 (67.6)	.01
Lack of professionals to prescribe PrEP	276 (57.4)	210 (62.0)	66 (46.5)	<.01
Limited time to discuss PrEP	171 (35.6)	111 (32.7)	60 (42.3)	.05
I have no knowledge about PrEP	165 (34.3)	89 (26.3)	76 (53.5)	<.001
I do not know where to refer a potential PrEP user^c^	81 (16.8)	81 (23.9)	–	N/A^d^

^a^Chi-square test

^b^Antiretroviral

^c^This question was not asked in Mexico as PrEP is not current public policy

^d^*N/A* Not applicable

## Discussion

Our results describe awareness, knowledge, and attitudes related to PrEP and other prevention strategies among HIV physicians from Brazil and Mexico and compare the differences between countries. Although awareness was similar in both countries, willingness to prescribe PrEP was higher in Brazil than Mexico, while barriers and concerns were more frequent in Mexico, which may be explained by the different stages of PrEP implementation in both countries. Awareness and willingness to prescribe PrEP (84.6% & 74.2%) were higher than previous studies conducted in LAC in Guatemala City (69% & 87%) and São Paulo, Brazil (75% & 63–69%) [26, 27], possibly due to increased information about PrEP over time and PrEP implementation in the Brazilian SUS since December 2017 [13]. Our results offer updated information to inform the Ministry of Health, stakeholders, clinicians and policy makers from Brazil, Mexico and other LAC countries on different stages of PrEP implementation [33].

Awareness of all other HIV prevention strategies except daily oral PrEP, including new PrEP technologies, was higher in Brazil than in Mexico [29]. A broader dissemination of information on combination HIV prevention including all available strategies is needed among Mexican HIV physicians to increase their knowledge beyond condoms, PEP, or PrEP. Increasing awareness and knowledge of new PrEP technologies under development or recently approved by regulatory agencies, such as cabotegravir injection [30] among healthcare workers in addition to HIV physicians could impact the acceptability and willingness to prescribe these technologies when they become available. Furthermore, almost all HIV physicians reported feeling very comfortable providing HIV/STI prevention counseling and performing clinical activities required for PrEP screening in clinical facilities [21] reflecting a positive attitude towards PrEP.

Although both countries established PEP policies more than 10 years ago [34], previous experience with this prevention strategy was more frequent among Brazilians. Over three-quarters of Brazilian HIV physicians previously prescribed PEP for all evaluated reasons for PEP use (occupational, consensual sex and sexual violence). Conversely, almost half of Mexicans prescribed PEP for occupational and sexual violence and only 26% for consensual sex. These results are worrisome and may indicate stigma and judgement by HIV physicians concerning sexual behavior. Continuous refreshing training and campaigns among Mexican HIV physicians should focus on recommending that PEP should be offered to all individuals with PEP criteria regardless of the reason of HIV exposure, and address physicians’ beliefs.

The proportion of Mexican HIV physicians trained in U=U was higher compared to Brazilians, although a lower proportion of Mexicans perceived the U=U slogan to be accurate. Still, proportions of HIV physicians perceiving U=U as accurate in both countries were still low considering the available scientific evidence of treatment as prevention [35–37] and efforts to increase U=U slogan more broadly since 2018 [38]. Reasons for health providers including HIV physicians to not fully embrace the U=U concept were persistent lack of trust and confidence, and a tendency to withhold the U=U slogan during counseling or clinical visits [39]. Either disbelief, concerns about risk compensation, or stigma towards stereotyped sexual behavior among people living with HIV usually result in a conservative message regarding U=U [40, 41]. Wider dissemination of the protective and preventive power of the U=U slogan among HIV physicians could improve their confidence in conveying this message to their patients living with HIV and help reduce the HIV-related stigma [31].

Brazilian and Mexican HIV physicians agreed that MSM would benefit from PrEP, possibly related to the high rates of HIV prevalence among this population [42]. However, it is worrisome that a relative low proportion of Mexicans did not consider that PrEP would benefit sex workers, transgender people, partners in sero-discordant relationships, and substance users, all populations at increased vulnerability for HIV infection in Mexico [43]. This could be a reflection of HIV physicians’ beliefs that these populations would not have the ability to adhere to daily oral PrEP and be retained at the clinic for follow-up visits [21]. For example, HIV physicians may delay ART initiation among substance users with recent HIV diagnosis due to their perception of substance users’ diminished ability to adhere to the treatment [44–46]. The same rational could be used by HIV physicians prescribing PrEP to such populations. However, in a recent study conducted in Brazil, transgender women showed high rates of retention after 1 year of PrEP provision and this was attributed to the gender-affirming setting [3]. Welcoming services to the most vulnerable populations may not only increase their retention but also HIV physicians’ perception on who can benefit from PrEP. Lastly, lower proportion of Brazilians considering that PrEP should be beneficial for any population may be related to high awareness of PrEP recommendations in Brazil, as PrEP is cost-effective only when offered to populations with HIV incidence higher than or equal to 3% [6].

Less than half of all the HIV physicians in both countries indicated that primary care and family clinics should offer PrEP, in contrast to some studies showing that HIV physicians believe that primary or family care physicians should prescribe PrEP because they have more HIV-uninfected patients (*purview paradox*) [23]. In our sample, the majority felt that specialized or HIV/STI clinics would be the most appropriate setting to offer PrEP, probably due to the belief that the physicians in these clinics have more experience prescribing ARV. It is striking that a large proportion of Mexicans agreed that behavioral interventions should be prioritized instead of PrEP use. Increasing the information on the efficacy of behavioral interventions in reducing HIV risk among vulnerable populations compared to the efficacy of PrEP could address this perception bias [47]. Concerns about budget reductions for ART if PrEP were available has been reported in both high- and low-income settings [20, 26].

Perceived barriers and concerns found in our study were similar to those reported by other studies in countries with PrEP availability [21, 22, 26, 27]. In general, ARV resistance or increased sexual risk behavior are common concerns among physicians who can prescribe PrEP to vulnerable populations [21]. However, barriers and concerns were more frequent among Mexicans in 13 of 15 items evaluated here, especially those related to PrEP medication and its daily use. Only those related to the public health system (consistent provision of PrEP and lack of professionals to prescribe PrEP) were higher among Brazilian HIV physicians, reflecting the concerns of physicians in countries where PrEP is already a public health policy. The recent decision to extend PrEP prescriptions to nurses in Brazil may reduce the concern related to available personnel for prescriptions [48].

### Strengths & limitations

As strengths, our study is the first to assess the awareness, knowledge, experience, and attitudes related to PrEP and other prevention strategies among HIV physicians from Brazil and Mexico, after these countries approved PrEP as a public health policy or conducted an implementation project. In addition, our results show that the perceived barriers or concerns to prescribing PrEP among physicians are different according to the stage of implementation, so there is a need to address these differences. Conversely, our study has some limitations. The cross-sectional design cannot identify associations or causality between the perceived barriers or concerns and the willingness to prescribe PrEP. We did not assess physicians’ sexual orientation which might have influenced their willingness to prescribe PrEP for key populations, such as MSM or TW. Also, this was a convenience sample, and our results cannot be generalized to all physicians from both countries, including the self-reported collection of data that could be subject to social desirability bias. We had a loss of 32% of participants who accessed and did not complete the survey, which could represent a lack of interest in PrEP or other prevention HIV strategies.

## Conclusions

In countries where the HIV epidemic is concentrated among historically stigmatized populations, such as LAC, combination HIV prevention strategies offer an opportunity to reduce new HIV infections. In addition to promoting PrEP use and other HIV prevention strategies among populations vulnerable to HIV, training, awareness-raising, and promotion of prescribing among physicians should also be strengthened. Depending on the stage of PrEP implementation, physicians may perceive obstacles related to the provision of services or lack of information to prescribe PrEP. These barriers should be addressed by HIV national programs in LAC to increase the number of people using HIV prevention technologies, contributing to the goal of ending the HIV epidemic by 2030.

## Supplementary Information

**Additional file 1.** Dataset PrEP Survey with Physicians from Brazil and Mexico 2020. This file contains the raw data from a survey performed in 2020 about the awareness, knowledge, and attitudes related to pre-exposure prophylaxis and other prevention strategies among physicians from Brazil and Mexico.

## Data Availability

All data generated or analyzed during this study are included in this published article and its supplementary information files.

## Abbreviations

ARV: Antiretroviral
CENSIDA (in Spanish): Mexican National HIV Program
ImPrEP: Implementation PrEP project
LAC: Latin America and the Caribbean
MSM: Men who have sex with men
PEP: Post-exposure prophylaxis
PrEP: Pre-exposure prophylaxis
STI: Sexual transmitted infections
SUS (in Portuguese): Brazilian Unified Health System
TAF/FTC: Tenofovir alafenamide and emtricitabine
TDF/FTC: Tenofovir disoproxil fumarate and emtricitabine
TW: Transgender women
U=U: Undetectable equals untransmittable
